# 3D-Printed Gastroretentive Floating Modified-Release Device for Dual Cardiovascular Drug Delivery: Isosorbide Dinitrate and Hydralazine

**DOI:** 10.3390/pharmaceutics18101278

**Published:** 2026-10-09

**Authors:** Dareena Jaiseri, Supusson Pengnam, Praneet Opanasopit, Phuvamin Suriyaamporn, Chaiyakarn Pornpitchanarong, Theerasak Rojanarata, Boonnada Pamornpathomkul, Prasopchai Patrojanasophon, Teeratas Kansom, Thapakorn Charoenying

**Affiliations:** 1 Pharmaceutical Development of Green Innovations Group (PDGIG) and Division of Industrial Pharmacy, Faculty of Pharmacy, Silpakorn University, Nakhon Pathom 73000, Thailand; 2 Pharmaceutical Development of Green Innovations Group (PDGIG) and Division of Biopharmaceutical Sciences and Pharmacology, Faculty of Pharmacy, Silpakorn University, Nakhon Pathom 73000, Thailand; 3 Research and Innovation Center for Advanced Therapy Medicinal Products, Faculty of Pharmacy, Silpakorn University, Nakhon Pathom 73000, Thailand

**Keywords:** cardiovascular drugs, gastroretentive floating drug delivery systems, hydralazine, isosorbide dinitrate, 3D printing

## Abstract

**Background**: Isosorbide dinitrate (ISDN) and hydralazine hydrochloride (HDZ) are dual cardiovascular drugs used to treat heart failure. However, the drugs have limitations such as short plasma half-life, low bioavailability, and potential for tolerance of the nitrate component. To address the need for prolonged drug release, a floating dual-compartment drug delivery system was developed to provide controlled drug release under simulated gastric dissolution conditions. **Objectives**: This study aimed to develop floating dual-compartment devices (FDCDs) to contain ISDN and HDZ gel and modify drug release under simulated gastric conditions. **Methods**: The FDCD was designed in a cylindrical shape consisting of an air chamber on the upper part for floating ability, and two compartments for loaded drug gel and controlling the drug release rate. Fused deposition modeling (FDM) was selected to fabricate FDCDs using polylactic acid (PLA) filament. The ISDN formulation varied the ratio of polyvinylpyrrolidone (PVP) to achieve drug release for 12 h. The HDZ formulation was optimized by varying the ratio of PVP and hydroxypropyl methylcellulose (HPMC) to extend drug release at 24 h. **Results**: The shape dimension and weight variation in the FDCDs showed low standard deviation (SD). FDCDs exhibited immediate floating, resisted overturning, and demonstrated in vitro floating ability for more than 24 h. The formulation ISDN3 at a PVP: drug: water ratio of 5:2:3 exhibited complete release at 12 h, while the formulation of HDZ1 at an HPMC: PVP: drug: water ratio of 0.3:2.5:2:5.2 showed complete release within 24 h. The ISDN3- and HDZ1-loaded FDCDs demonstrated prolonged release behavior, with ISDN and HDZ release profiles showing the best fit to the zero-order and Higuchi models, respectively, based primarily on the coefficient of determination (R^2^), while root mean square error (RMSE) was used as a complementary measure of fitting error. **Conclusions**: These findings suggest that the developed dual-drug-loaded FDCD has potential as a dual-drug oral delivery platform for simultaneous delivery of ISDN and HDZ with prolonged drug release characteristics and further development of oral combination drug delivery systems.

## 1. Introduction

Gastroretentive floating drug delivery systems (GRFDDSs) are an approach to oral drug delivery systems that prolong residence time in the stomach based on the principle of buoyancy in gastric fluid, with a density lower than that of gastric fluid (1.004–1.010 g/mL) [1]. These systems increase bioavailability, extend gastric residence time, improve patient compliance, reduce drug side effects by maintaining consistent plasma drug concentrations, avoid poor intestinal drug absorption or rapid elimination, and provide local treatment of the stomach [2,3,4]. Currently, three-dimensional (3D) printing technology is an innovative drug delivery strategy for improving the effectiveness of GRFDDSs, enabling the design of complex geometries with customized release profiles for personalized therapeutics [5,6,7,8].

3D printing technology is a novel technique that has been extensively researched across various fields for the manufacture of 3D objects [9]. In the pharmaceutical field, research interest has been growing since 2015, after the U.S. Food and Drug Administration (FDA) approved the first oral, fast-dissolving 3D-printed tablets (Spritam^®^), manufactured using a binder-jetting 3D printer [10]. 3D printing has been applied to fabricate solid dosage forms with diverse shapes, geometries, and sizes, enabling on-demand production and allowing adjustment of drug release characteristics [5,11,12,13,14]. Fused deposition modeling (FDM) 3D printers are widely studied for pharmaceutical applications due to their cost-effectiveness, high accuracy, high resolution, ease of use, and the absence of post-processing steps, among other factors [11,15]. FDM has been utilized in the design of GRFDDSs through two approaches. The first approach uses drug-loaded filaments, which are printed tablets that have low-density structures for controlling drug release. However, this technique is unsuitable for drugs that are sensitive to high temperatures because FDM relies on the high temperatures of the nozzle to extrude materials and create 3D objects [16,17,18]. The second approach employs non-drug-loaded filaments to fabricate hollow structures with an air cavity, and containing the traditional dosage form in separate compartments [4,19,20,21,22,23,24]. As reported by Alqahtani et al., a GRFDDS dual-compartment device was fabricated by FDM 3D printing using non-drug-loaded filament. The commercial propranolol hydrochloride immediate-release tablet was placed into the inner compartment, while the outer compartment served as the air chamber. The drug was released through a smaller window with varying sizes (1–4 mm). The results showed that the devices printed with commercial polyvinyl alcohol (PVA) filament achieved complete drug release and had a total floating time of approximately 3 h. In contrast, the polylactic acid (PLA) commercial filament allowed the device to float for more than 24 h due to the hydrophobic structure, with sustained drug release for over 10 h [25]. Jaiseri et al. produced floating devices for incorporating a propranolol gel formulation using FDM 3D printing with a commercial PLA filament. The device was designed to include an air cavity for buoyancy, and a varying number of open channels to contain the propranolol gel, and enable modified release. The results demonstrated that the floating device exhibited buoyancy for more than 24 h and sustained drug release for 8 h through two and three channels. Furthermore, the 3D-printed device filled with drug gel enables simple adjustments to the drug potency for individualized treatment [26]. Maroni et al. fabricated two-compartment devices for dual-drug release *via* FDM 3D printing with non-drug-loaded filaments. The device was designed with compartments having different wall thicknesses (600 or 1200 μm). The results indicated that the devices exhibited a two-pulse release pattern, which correlated with the properties of the filament material and wall thickness in determining release duration [27]. Thus, FDM 3D printing technology overcomes the limitations of traditional drug manufacturing. The design model, type of filament material, and drug dosage form are key factors that influence the drug release characteristics.

Isosorbide dinitrate (ISDN) and hydralazine (HDZ) are used in combination for the treatment of heart failure, with demonstrated benefits in reducing morbidity and mortality [28,29]. In addition, the combination of ISDN and HDZ is considered an alternative therapeutic option for patients who are intolerant of angiotensin-converting enzyme inhibitors (ACEIs) or angiotensin II receptor blockers (ARBs) [30]. Currently, BiDil^®^ is a commercial immediate-release tablet containing 20 mg of ISDN and 37.5 mg of HDZ approved by the U.S. FDA. The recommended starting dose is one tablet three times a day, with a potential increase to two tablets three times a day based on patient tolerance [31]. In clinical studies, the treatment regimen has also been initiated at a lower dose of one tablet twice daily, followed by dose titration according to patient tolerance and clinical response [32]. However, the limitations of BiDil^®^ include side effects, potential for tolerance due to its nitrate component, a short plasma half-life (1–4 h), and low bioavailability due to high first-pass hepatic metabolism [31,33,34,35]. HDZ, a weakly basic drug with a reported pKa of approximately 7.3 [36,37], undergoes protonation under acidic conditions. Consequently, the gastric environment may promote the presence of the ionized form of hydralazine, which is generally associated with improved aqueous solubility of weak bases. Therefore, a gastroretentive system may provide a favorable environment for sustained release of hydralazine through prolonged gastric residence and controlled drug diffusion. Therefore, ISDN and HDZ were selected as model drugs for developing a floating dual-compartment drug delivery system. The FDCD fabricated by FDM 3D printing with drug-loaded gel formulations was designed to provide controlled release of both drugs while maintaining their individual release characteristics.

The aim of this study was to develop floating dual-compartment devices (FDCDs) containing ISDN and HDZ gel formulations to modify in vitro drug release using gel formulations with different polymer ratios. The rationale for developing a floating dual-compartment system for ISDN and HDZ is based on the need to provide prolonged and controlled delivery of both drugs while maintaining their individual release characteristics. The dual-compartment design enables separate control of each drug gel formulation, allowing ISDN and HDZ to be simultaneously delivered from a single device. The novelty of this approach lies in integrating drug-specific gel formulations with individually tailored release characteristics into a single FDCD platform for simultaneous delivery of ISDN and HDZ. The FDCD was designed to have two compartments on the device’s lateral wall to contain the drug gels, with an air cavity in the upper part of the FDCD to enhance floating. An FDM 3D printer was selected to print the FDCD using commercial PLA filament due to its water-insoluble properties, which help maintain the structural integrity of the device and support floating ability. The drug gel formulations consisted of two groups: the ISDN gel was prepared to achieve release in 12 h, and the HDZ gel was formulated for complete release in 24 h. The weight variation, morphology, anti-flip-up performance, and floating time of drug-loaded FDCDs were evaluated. In vitro drug release profiles and kinetics of release of ISDN and HDZ from FDCDs with different gel formulation ratios were analyzed.

## 2. Materials and Methods

### 2.1. Materials

ISDN powder was donated by the Government Pharmaceutical Organization (GPO) (Bangkok, Thailand). Commercial ISDN (10 mg) tablets were purchased from Siam Bheasach Co., Ltd. (Bangkok, Thailand). HDZ HCl (50 mg) tablets were obtained from T.O. Pharma Co., Ltd. (Samut Prakan, Thailand). HDZ HCl powder, polyvinylpyrrolidone (PVP K-90) MW of approximately 1.3 × 10^6^, and sodium chloride (NaCl) were purchased from Sigma-Aldrich (Steinheim, Germany). Hydroxypropyl methylcellulose (HPMC) (METHOCEL™ F4M, viscosity range 3500–5600 mPa·s in a 2% solution) was purchased from P.C. Drug Center Co., Ltd. (Bangkok, Thailand) (Batch No. F410KCUC42). Methanol and 37% (*w*/*w*) HCl were obtained from Merck KGaA (Darmstadt, Germany). Commercial PLA+ filament (diameter 1.75 mm) was obtained from eSun Industrial Co., Ltd. (Shenzhen, China).

### 2.2. Design and FDM Printing of the FDCD

**Figure 1:**
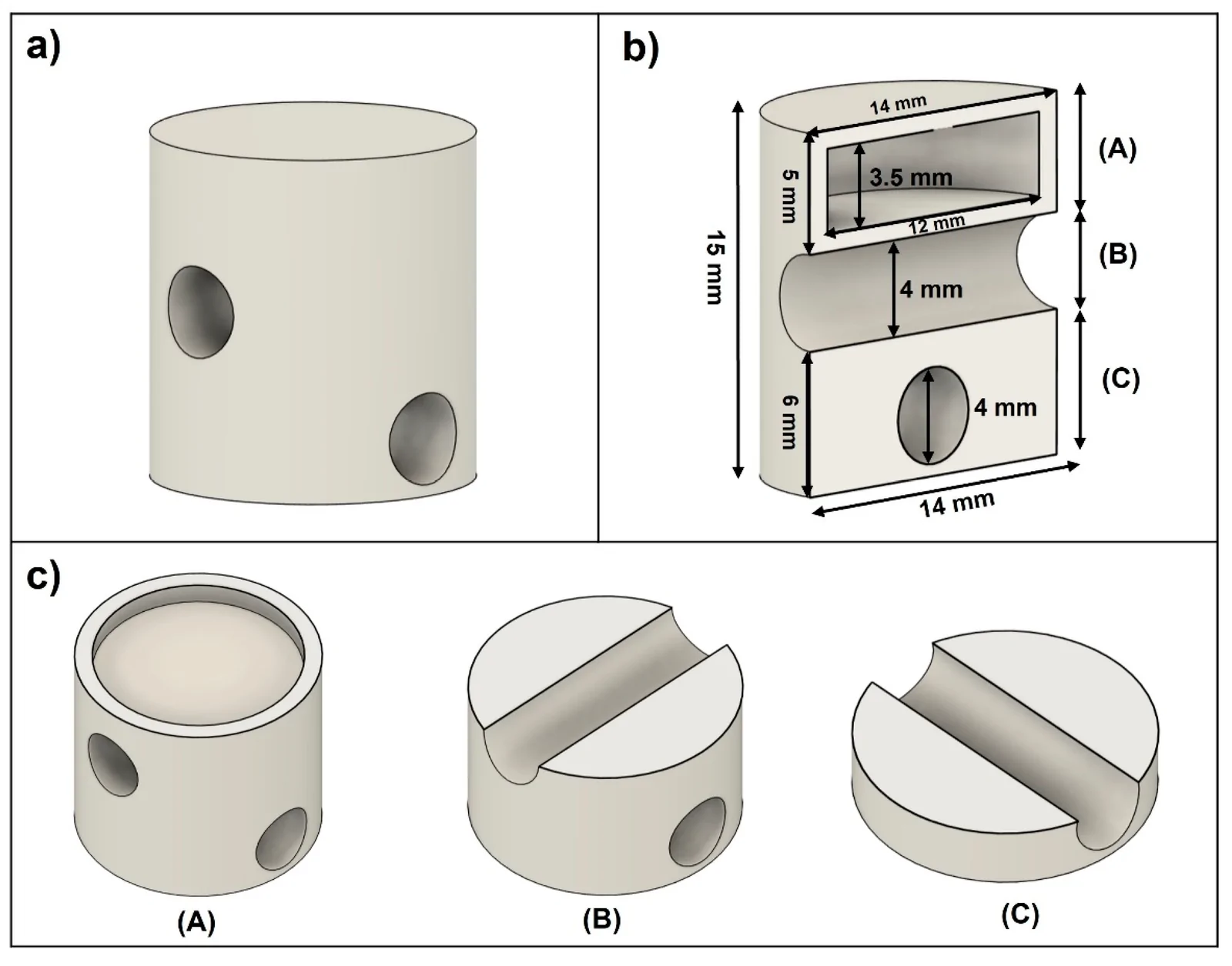
Design and dimensions of the FDCD. (**a**) Three-dimensional model of the FDCD. (**b**) Vertical cross-sectional view showing the locations of (**A**) the first compartment (air chamber), (**B**) the second compartment for ISDN gel loading, and (**C**) the third compartment for HDZ gel loading. (**c**) Horizontal cross-sectional views showing the internal structure of the FDCD, including the air chamber (**A**), ISDN gel-loading compartment (**B**), and HDZ gel-loading compartment (**C**).

The FDCD model was designed using Autodesk^®^ Fusion 360^TM^ Student software version 2.0.7819 (Autodesk, Inc., San Francisco, CA, USA). The designed file was imported into Bambu Studio software version 1.8.4.51 (Bambu Lab, Shenzhen, China) for slicing the model and adjusting the 3D printer settings. The FDCD was designed in a cylindrical shape consisting of three parts: the first compartment, an air chamber located at the upper part of the tablet to provide floating ability; the second compartment; and the third compartment, which contained the drug gel. The compartments of the 3D printing tablet were divided into two compartments, each with a diameter of 4 mm, to contain ISDN gel and HDZ gel, respectively, and to control the drug release rates. The dimensions of the FDCD are presented in Figure 1. The 3D-printed FDCD was fabricated using a Bambu Lab X1 Carbon FDM 3D printer (Bambu Lab, Shenzhen, China) with a nozzle diameter of 0.4 mm, and PLA commercial filaments (white color, and a diameter of 1.75 mm). The 3D printer was modified by adjusting several parameters, including an infill percentage of 15%, a rectilinear infill pattern, and an infill layer height of 200 µm. The extruder speed was set to 45 mm/s during extrusion and 180 mm/s during travel. The build plate and extruder head temperatures were maintained at 45 °C and 215 °C, respectively.

### 2.3. Physical Characterizations of FDCDs

The morphology of the FDCD was investigated using a digital microscope (AnMo Electronics Corporation, New Taipei City, Taiwan). The dimensions of the FDCD were measured using a digital caliper (Zhejiang Deqing Syntek Electronic Technology Co., Ltd., Deqing, China). Twenty devices were weighed individually to determine the accurate weight of each tablet using an analytical balance Sartorius^®^ series-CP224S (Data Weighing Systems, Inc., Wood Dale, IL, USA). The results were reported as the mean ± standard deviation (SD) of the twenty independent devices in mg (*n* = 20).

### 2.4. Preparation and Rheological Characterization of Base Gel Formulations

The polymer-based gel formulations were prepared using PVP and HPMC as gel-forming polymers with deionized (DI) water, excluding the active pharmaceutical ingredients. The composition of the base gel formulations is presented in Table 1. The prepared gel formulations were mixed until homogeneous gels were obtained. The physical appearance of each base gel formulation was observed and recorded. The rheological properties of the base gel formulations were evaluated using a rotational rheometer (DSR Malvern–Kinexus Pro, Malvern Instruments, Malvern, UK). The measurements were performed at 37 °C under controlled temperature conditions to simulate physiological conditions in the human body. The flow behavior of each formulation was characterized using a shear rate ramp test. The shear stress and apparent viscosity were recorded over the applied shear rate range. The flow behavior of the gel formulations was evaluated by plotting shear stress as a function of shear rate.

### 2.5. Preparation of Drug Gel Formulations and Loading into FDCDs

The drug gel formulations were prepared by individually mixing the drug with a water-soluble polymer and DI water. The resulting mixtures were then placed in a vacuum chamber for 3 h to eliminate air bubbles. The composition of gel formulations is shown in Table 2. The drug gel formulations were loaded into the designated compartments of the FDCD based on the calculated amount required to achieve the desired drug dose. The ISDN gel formulation (200 mg), containing 40 mg of ISDN, was loaded into the second compartment, whereas the HDZ gel formulation (187.50 mg), containing 37.5 mg of HDZ, was loaded into the third compartment. The weight of the drug gel-loaded FDCDs was determined by weighing ten individual FDCDs using a digital balance (*n* = 10). The reproducibility of the gel-loading process was evaluated based on the variation in gel-loading weight and expressed as percentage relative standard deviation (%RSD). The drug gel-loaded FDCDs were dried at 60 °C in a hot-air oven for 12 h to remove the moisture, followed by the next step of the experiment.

### 2.6. Anti-Flip-Up Evaluation

The anti-flip-up investigation aimed to determine the ability of the floating drug delivery systems to maintain the FDCD position under simulated gastric conditions without flipping after administration. A drug gel-loaded FDCD was placed into an Erlenmeyer flask containing simulated gastric fluid (SGF) at pH 1.2 to mimic the acidic environment of the stomach. Next, the flask was placed on a shaker set at a speed of 50 rpm for 30 min, then the flask was closed and inverted a total of 10 times, with observations made to determine the number of times the device flipped. The anti-flip-up test was performed using three independent FDCDs for each formulation (*n* = 3), and the results were reported as the percentage of devices that maintained their original orientation without flipping.

### 2.7. Analytical Methods for ISDN and HDZ Quantification

The analytical methods for single-drug quantification were evaluated prior to the analysis of drug content and release samples obtained from FDCDs. ISDN was quantified using a high-performance liquid chromatography (HPLC) method, whereas HDZ was quantified using a UV–visible spectrophotometric method.

The ISDN content was measured using high-performance liquid chromatography (HPLC) adapted from the method reported by Madhu et al. with modifications [38]. The analysis was performed using an Agilent 1260 Infinity Series (Agilent Technologies Inc., Santa Clara, CA, USA) equipped with a Shiseido reversed-phase column 18 (4.6 mm × 250 mm, 5 µm particle size) (Tokyo, Japan). The column temperature was maintained at 25 °C. The injection volume was 20 µL. The detector was set to UV at a wavelength of 214 nm. The mobile phase consisted of methanol and a 0.1 M aqueous ammonium sulfate solution (50:50, *v*/*v*). Chromatographic separation was performed under isocratic conditions at a flow rate of 1.0 mL/min. The run time was set to 6 min. Standard ISDN solutions were prepared at concentrations ranging from 1.56–100 µg/mL to construct the calibration curve. The HDZ content was quantified using UV–visible spectrophotometry. The analysis was performed using a VICTOR^®^ Nivo™ multimode microplate reader (PerkinElmer, Waltham, MA, USA) at a wavelength of 260 nm. Standard HDZ solutions were prepared at concentrations ranging from 10–110 µg/mL.

The analytical performance of both methods was evaluated using individual drug standard solutions. Linearity was assessed based on the coefficient of determination (R^2^), slope, and intercept of the calibration curves. Accuracy was evaluated by recovery studies at three different concentration levels, and precision was determined by six measurements analysis (*n* = 6). The results were expressed as percentage recovery and %RSD.

### 2.8. Screening of In Vitro Floating Ability and Drug Release Profiles of Single-Drug-Loaded FDCDs

The single-drug-loaded FDCDs were initially screened to identify the ISDN and HDZ gel formulations that achieved the desired release durations of approximately 12 h and 24 h, respectively. The selected formulations with release profiles closest to the target durations were subsequently used for the development of dual-drug-loaded FDCDs. The dissolution tester apparatus 2 (DT 720, ERWEKA GmbH, Heusenstamm, Germany) was used to evaluate the floating properties and drug release profiles of the drug gel-loaded FDCDs under conditions of 37 ± 0.5 °C, using 900 mL of SGF (0.1 N HCl) at pH 1.2, and a paddle rotation speed of 75 rpm. The floating lag time and the total duration of floating of the drug gel-loaded FDCDs were measured. The in vitro drug release profiles of ISDN and HDZ-loaded FDCDs were investigated at various time points of 5, 15, and 30 min, and at 1, 2, 4, 6, 8, 12, and 24 h. At each time point, 2 mL of sample was withdrawn from the dissolution medium. Then, the volume of the medium was maintained by immediately adding 2 mL of fresh medium to replace the withdrawn sample. The sample was filtered using a 0.45 μm nylon filter. The concentrations of ISDN and HDZ in the dissolution samples were determined using the analytical methods described in Section 2.7.

### 2.9. Evaluation of the Analytical Method for Simultaneous Quantification of ISDN and HDZ in Dual-Drug-Loaded FDCDs

The HPLC analytical method for simultaneous quantification of ISDN and HDZ was adapted from a previously validated method reported in [39,40]. The analytical performance was evaluated based on specificity, linearity, accuracy, precision, and recovery. Specificity was evaluated by comparing the chromatograms of blank dissolution medium, placebo gel containing PVP and HPMC without drug, and ISDN and HDZ standard solutions. The absence of interfering peaks at the retention times (RTs) of ISDN and HDZ confirmed the specificity of the analytical method. The ISDN and HDZ concentrations were quantified using HPLC equipped with an Agilent 1260 Infinity Series system and an Agilent C18 reversed-phase column (250 mm × 4.6 mm, 5 µm particle size) (Agilent Technologies Inc., CA, USA). The column temperature was maintained at 25 °C. The HPLC analysis was performed using a gradient elution program. The mobile phase consisted of three components: 0.01 M ammonium acetate buffer (pH 3), acetonitrile, and methanol. The gradient elution program was performed according to the conditions shown in Table S3. The flow rate was set at 1 mL/min. The detection wavelengths were set at 214 nm for ISDN and 260 nm for HDZ. The injection volume was 20 µL. The linearity of the HPLC analytical method was evaluated by constructing calibration curves of ISDN and HDZ standard solutions prepared in SGF (0.1 N HCl, pH 1.2) over the concentration range of 1.56–100 µg/mL. The linear regression equations and R^2^ values were calculated. The accuracy of the analytical method was assessed by recovery testing at three concentration levels (12.5, 25, and 50 µg/mL). Each concentration level was analyzed in triplicate, and the percentage recovery was calculated. The precision of the method was evaluated at a concentration of 25 µg/mL using six measurements. The results were expressed as the %RSD of the peak areas for ISDN and HDZ.

### 2.10. Evaluation of Drug Content Uniformity and Drug Loss During Preparation of Dual-Drug-Loaded FDCDs

Based on the screening results, ISDN3 and HDZ1 gel formulations were selected and loaded into separate compartments of the FDCD for preparation of a dual-drug-loaded FDCD. The selected formulations were loaded into an FDCD for evaluation of drug content uniformity and drug loss during the preparation process. The drug content uniformity of ISDN3- and HDZ1-loaded FDCDs was evaluated by determining the ISDN and HDZ contents in individual FDCD. Ten individual dual-drug-loaded FDCDs were evaluated for drug content uniformity (*n* = 10). The dual-drug-loaded FDCD was dissolved in 900 mL of SGF (0.1 N HCl, pH 1.2) using a bath sonicator at 37 °C until complete drug release was achieved. The released ISDN and HDZ concentrations were quantified using the HPLC analytical method for simultaneous quantification of ISDN and HDZ described in Section 2.9. The content uniformity was expressed as the mean drug content and %RSD of individual FDCD. The drug loss during FDCD preparation was evaluated by comparing the initial drug content in the gel formulations before loading into the FDCD with the final drug content after the loading and drying processes. The initial drug content was determined based on the theoretical amount of ISDN and HDZ incorporated into the gel formulations. The final drug content was determined from the dual-drug-loaded FDCD after drying at 60 °C for 12 h. The percentage of drug loss was calculated using Equation (1):

(1) Drug loss (%) = Initial drug content − Final drug content Initial drug content  × 100

### 2.11. In Vitro Floating Ability and Dual-Drug Release Study of ISDN3- and HDZ1-Loaded FDCD

Based on the results of the single-drug-loaded FDCD screening study, ISDN3 and HDZ1 gel formulations were selected based on their release durations of approximately 12 h for ISDN and 24 h for HDZ, respectively. Therefore, these selected gel formulations were further evaluated in a dual-drug-loaded FDCD. The selected ISDN3 and HDZ1 gels were loaded into separate compartments of the same FDCD as described in Section 2.5. The floating ability of the dual-drug-loaded FDCD was evaluated under the same dissolution conditions described in Section 2.8. The floating lag time was recorded as the time required for the FDCD to rise to the surface of the dissolution medium after immersion. The total floating time was determined by visually monitoring the FDCD until the end of the dissolution study. The in vitro drug release study was performed under the dissolution conditions described in Section 2.8. Briefly, the dual-drug-loaded FDCD was evaluated using USP dissolution apparatus II containing 900 mL of SGF (0.1 N HCl, pH 1.2) at 37 ± 0.5 °C with a paddle rotation speed of 75 rpm. Dissolution samples were collected at predetermined time intervals of 5, 15, and 30 min, and 1, 2, 4, 6, 8, 12, and 24 h. The withdrawn samples were analyzed for simultaneous quantification of ISDN and HDZ using the HPLC analytical method described in Section 2.9.

### 2.12. Evaluation of Post-Dissolution Characteristics of ISDN3- and HDZ1-Loaded FDCDs

The physical changes in FDCDs before and after the dissolution study were evaluated. The external appearance of FDCDs was visually observed and photographed before and after dissolution to assess apparent structural changes, including swelling, deformation, cracking, and erosion. In addition, the diameter and height of FDCDs were measured using a digital vernier caliper before and after dissolution to evaluate dimensional changes during the dissolution period. The weight of each empty FDCD was measured before gel loading. After dissolution, all residual drug gel was removed, and the FDCD was oven-dried at 50 °C for 24 h and weighed again to evaluate changes in device weight. The percentage weight change in the PLA devices was calculated from the difference between the initial and final weights of the FDCDs. All measurements were performed using three independent devices (*n* = 3).

### 2.13. Drug Release Kinetics

The drug release kinetics were assessed to characterize the release profiles of the FDCD formulations. The kinetic models were applied to the drug release data obtained during the principal release phase. The fitting interval was selected individually for each formulation, beginning at the first sampling time point showing measurable drug release and extending to the last sampling point before a clear leveling-off of the cumulative release profile. When no plateau was observed within the experimental period, data up to the final available sampling point were included. The experimental release data were fitted to three mathematical models: zero-order, first-order, and Higuchi models. Model selection was based primarily on the R^2^, while root mean square error (RMSE) was used as a complementary measure of goodness of fit, particularly when R^2^ values were similar among candidate models. The selected kinetic models were interpreted as empirical descriptions of the observed release profiles and were not used as definitive evidence of the underlying drug release mechanisms.

### 2.14. Statistical Analysis

Unless otherwise stated, experiments were conducted using three independent device replicates (*n* = 3), and data are presented as the mean ± SD. Differences in drug content before and after the drying process were analyzed using a dependent *t*-test. Drug release profiles were analyzed using two-way analysis of variance (ANOVA) followed by Tukey’s honestly significant difference post hoc test for multiple comparisons. Single- and dual-drug release profiles were compared separately for each drug using two-way repeated-measures ANOVA, with loading configuration as the between-device factor and time as the within-device factor. Greenhouse–Geisser correction was applied to effects involving time. Significant interactions were followed by two-sided Welch’s *t*-tests with Holm adjustment across evaluable sampling times. Statistical significance was set at *p* < 0.05, and exact *p*-values are reported where applicable.

## 3. Results

### 3.1. Physical Appearance and Rheological Properties of Base Gel Formulations

The physical appearance of the base gel formulations is shown in Figure S1. ISDN1–ISDN3 and HDZ1–HDZ3 formulations exhibited different gel characteristics depending on the polymer composition. The ISDN formulations (ISDN1–ISDN3), containing increasing amounts of PVP, showed progressively increased gel consistency, whereas the HDZ formulations (HDZ1–HDZ3), prepared with increasing HPMC concentrations, exhibited visually different gel characteristics. These observations indicated that polymer composition influenced the structural characteristics of the gel matrices.

**Figure 2:**
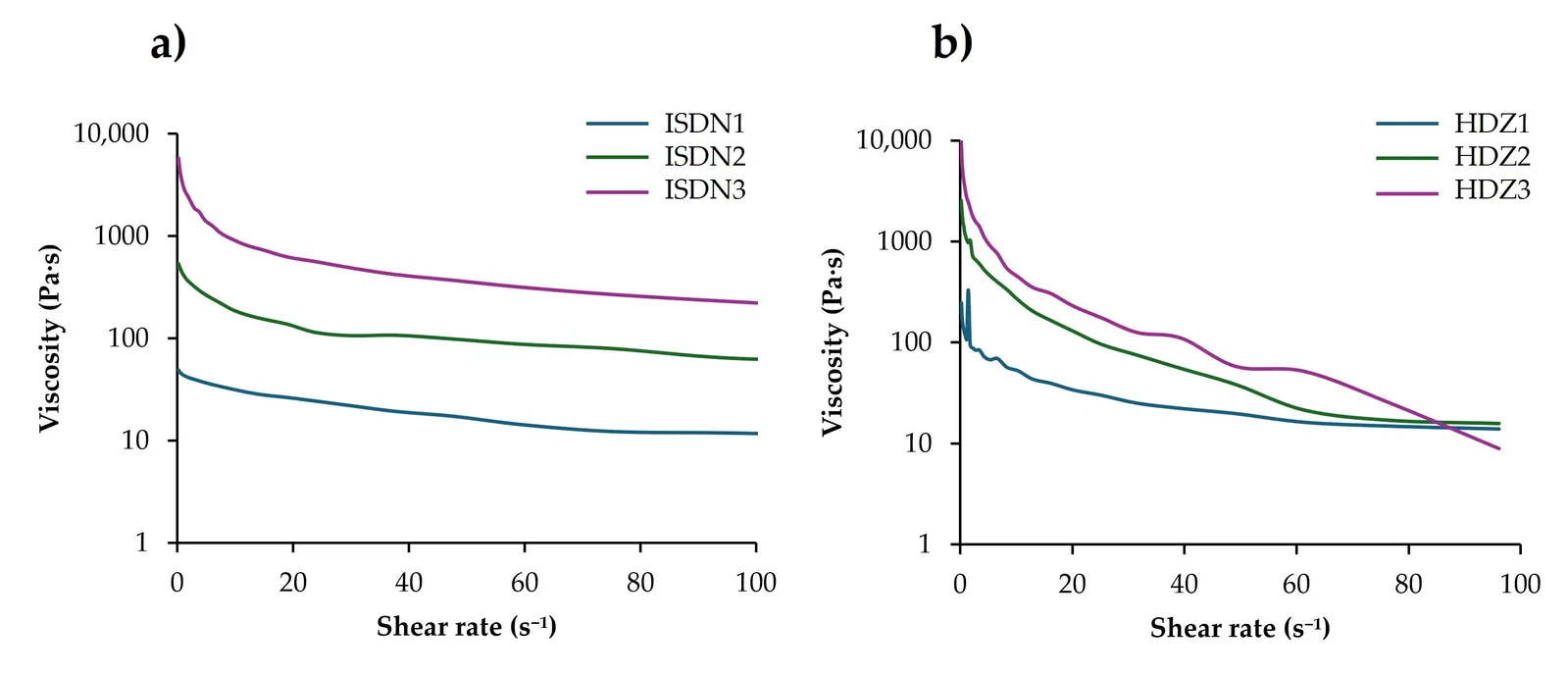
Apparent viscosity as a function of shear rate for (**a**) ISDN base gels and (**b**) HDZ base gels.

The rheological properties of the base gel formulations were evaluated by determining the apparent viscosity as a function of shear rate (Figure 2). All formulations exhibited a decrease in apparent viscosity with increasing shear rate, indicating shear-thinning behavior. This behavior is commonly observed in polymer-based gel systems and can be attributed to the rearrangement of polymer networks under applied shear. For the ISDN formulations, the apparent viscosity increased with increasing PVP concentration. ISDN3 exhibited the highest viscosity throughout the investigated shear rate range, followed by ISDN2 and ISDN1, suggesting that higher PVP concentrations contributed to increased resistance to flow and enhanced polymer network interactions. Similarly, the HDZ formulations showed increased apparent viscosity with increasing HPMC concentration. HDZ3 exhibited the highest viscosity, followed by HDZ2 and HDZ1, indicating that increased HPMC content increased the viscosity and resistance to flow of the gel matrix.

The corresponding shear stress–shear rate profiles further supported the differences in flow behavior among formulations (Figure S2). Higher polymer concentrations resulted in increased shear stress values at comparable shear rates, indicating greater resistance to flow. The differences in rheological properties may partially explain the variation in drug release behavior observed during subsequent dissolution studies.

### 3.2. Characterizations of FDCDs

**Figure 3:**
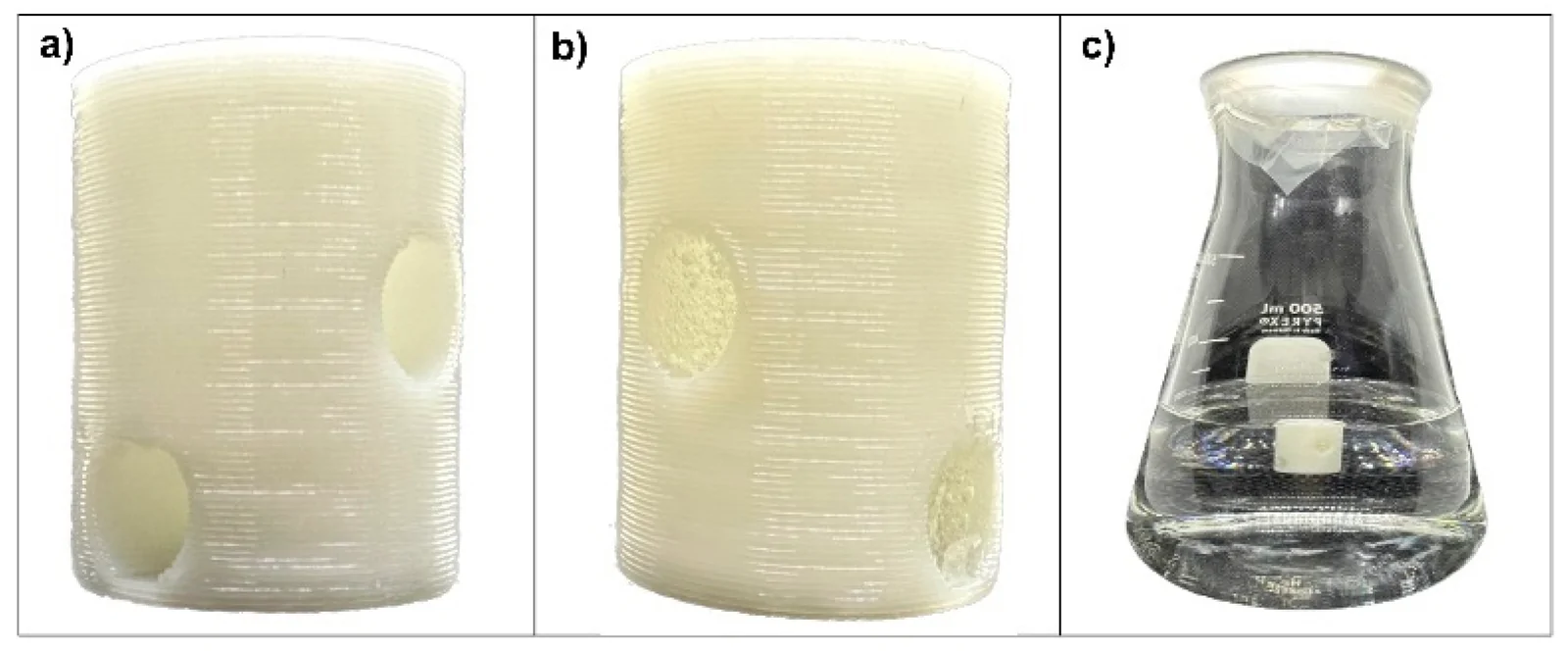
Photographs of (**a**) the FDCD, (**b**) ISDN- and HDZ-loaded FDCD, and (**c**) anti-flip-up evaluation of ISDN- and HDZ-loaded FDCD.

The FDCDs were produced using FDM with PLA. The appearance and morphology of the FDCDs were assessed using a digital microscope and are shown in Figure 3a. The white appearance of the FDCDs was attributed to the color of the PLA filament. A smooth surface was identified on the FDCDs. The ISDN and HDZ gels were loaded into the compartments of the FDCDs, and satisfactory characteristics of the ISDN- and HDZ-loaded FDCDs were demonstrated in Figure 3b. The FDCDs exhibited dimensions of 15.10 ± 0.02 mm in height, 13.78 ± 0.23 mm in diameter, and a compartment diameter of 3.79 ± 0.05 mm. The weight of the FDCDs had an average of 936.25 ± 1.25 mg. For the dual-drug-loaded FDCDs, nominal amounts of ISDN gel (200 mg) and HDZ gel (187.50 mg) were loaded into their respective compartments, corresponding to a total wet-gel mass of 387.5 mg. After drying, the final weight of the ISDN- and HDZ-loaded FDCDs was 1266.20 ± 2.70 mg, indicating low variability among the prepared devices. Based on the water fractions of the ISDN3 and HDZ1 gels, the theoretical completely dry mass was 1166.25 mg. The difference of approximately 100 mg between the theoretical completely dry mass and the measured post-drying mass may reflect residual moisture retained within the gel matrices after drying. However, because residual moisture was not independently measured, this explanation remains tentative, and the mass balance could not be conclusively verified.

### 3.3. Anti-Flip-Up of ISDN- and HDZ-Loaded FDCDs

The anti-flip-up of the ISDN and HDZ-loaded FDCDs was evaluated using shaking on a shaker machine and upside-down turning. The FDCDs resisted overturning due to the presence of an air chamber incorporated into the upper part of the device; this is illustrated in Figure 3c. All tested formulations maintained their original orientation without flipping after 10 inversion cycles, resulting in a 100% anti-flip-up success rate.

### 3.4. Analytical Performance of ISDN and HDZ Quantification Methods

The analytical performance of the HPLC method for ISDN quantification and the UV–visible spectrophotometric method for HDZ quantification was evaluated based on linearity, accuracy, and precision. The ISDN peak was observed at an RT of approximately 5.03 min. The calibration curves for ISDN (1.56–100 µg/mL) and HDZ (10–110 µg/mL) yielded R^2^ values of 0.9993 and 0.9996, respectively. The analytical performance parameters are summarized in Tables S1 and S2.

### 3.5. In Vitro Floating Ability and Drug Release from Single-Drug-Loaded FDCDs

**Figure 4:**
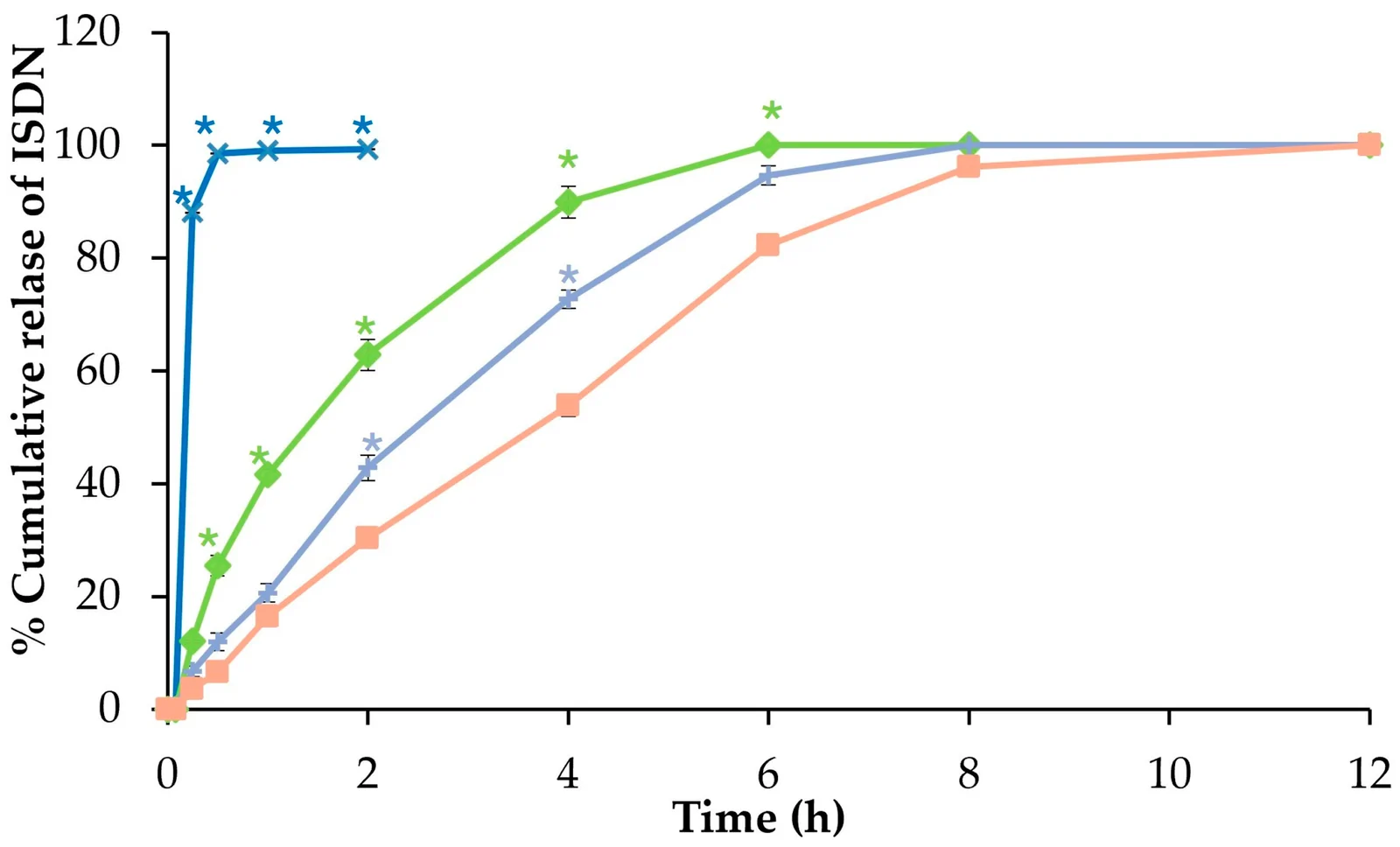
In vitro drug release from commercial immediate-release ISDN tablets (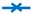), and ISDN-loaded FDCDs with ISDN1 (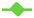), ISDN2 (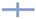), and ISDN3 (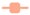). * Statistically significantly (*p* < 0.05) higher than ISDN3. All measurements were performed with three independent devices (*n* = 3).

**Figure 5:**
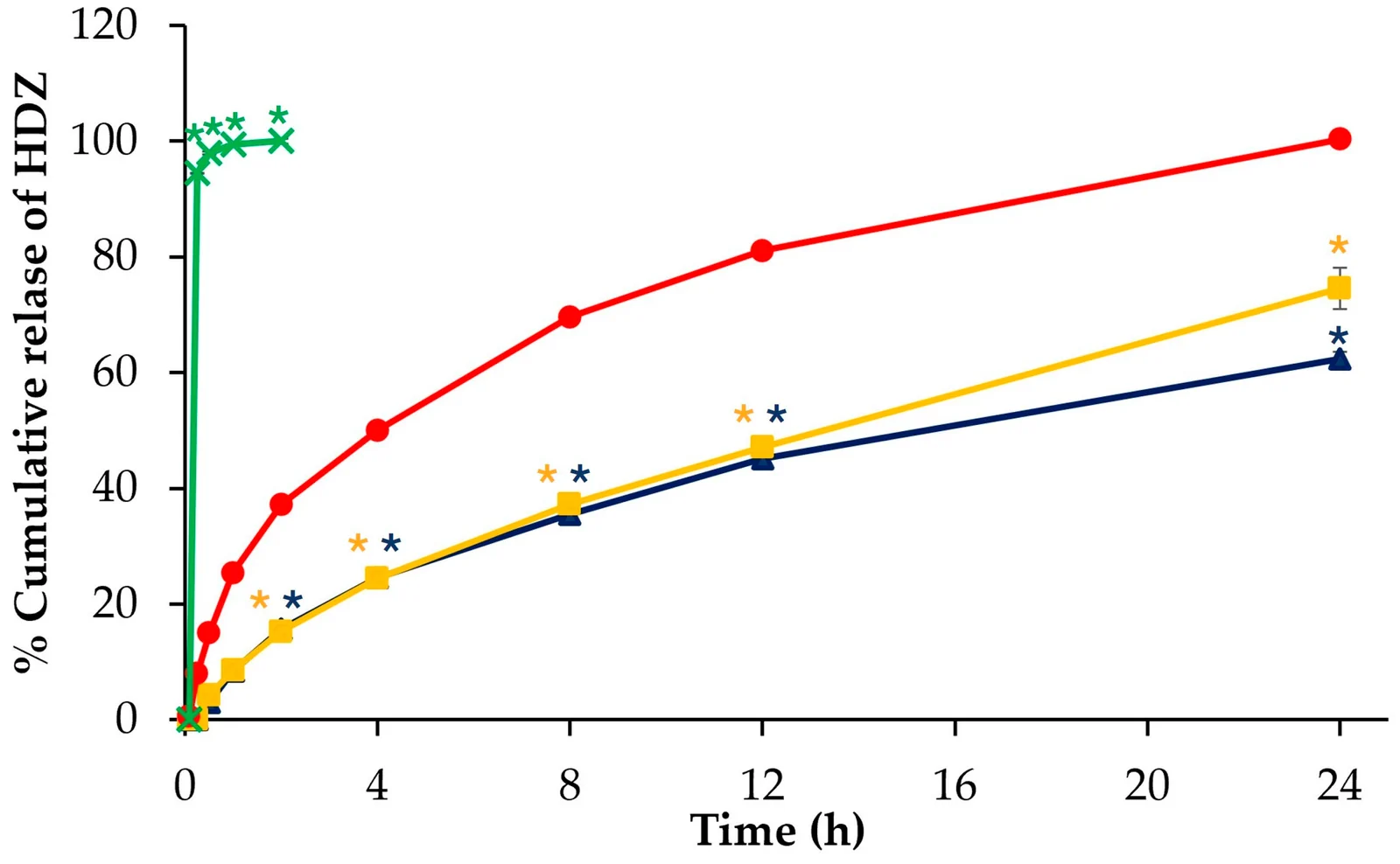
In vitro drug release from commercial immediate-release HDZ tablets (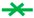), and HDZ-loaded FDCDs with HDZ1 (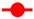), HDZ2 (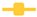), and HDZ3 (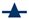) * Statistically significantly different (*p* < 0.05) compared with HDZ1. All measurements were performed with three independent devices (*n* = 3).

The ISDN- and HDZ-loaded FDCDs demonstrated immediate floating ability in SGF at pH 1.2, with a floating lag time of 0 min and maintained buoyancy for more than 24 h. The release profiles of ISDN and HDZ from various formulations in FDCDs exhibited sustained release. The formulation of ISDN3 showed a slow release rate, reaching more than 80% cumulative release after 6 h, and was completely released in 12 h. In comparison, the ISDN1 and ISDN2 gel formulations showed the faster release profile, with more than 95% cumulative release at 6 h, as shown in Figure 4. In addition, the formulation of HDZ1 presented a fastest release rate, achieving more than 80% cumulative release after 12 h, and was completely released in 24 h. On the other hand, the gel formulations of HDZ2 and HDZ3 showed a slower release profile of approximately 45% cumulative release at 12 h. Still, they did not achieve complete release within 24 h. The results, presented in Figure 5. The dissolution of ISDN (10 mg) and HDZ (50 mg) commercial immediate-release tablets showed an immediate release profile and was completely released within 30 min (Figure 4 and Figure 5).

### 3.6. Analytical Performance of the HPLC Method for Simultaneous Quantification of ISDN and HDZ in Dual-Drug-Loaded FDCDs

The specificity of the HPLC analytical method was evaluated by comparing the chromatograms of blank dissolution medium, placebo gel containing PVP and HPMC without a drug, and ISDN and HDZ standard solutions. No interfering peaks were observed at the RT of ISDN and HDZ, indicating that the developed HPLC method was specific for simultaneous quantification of ISDN and HDZ in the presence of formulation components. The ISDN and HDZ peaks were identified at RTs of approximately 13.33 and 2.69 min, respectively. The linearity of the analytical method was evaluated over the concentration range of 1.56–100 µg/mL for both ISDN and HDZ. The calibration curves showed good linear relationships between peak area and drug concentration, with R^2^ values of 0.9993 for ISDN and 0.9999 for HDZ (Figures S5 and S6). The regression equations and calibration parameters are summarized in Table 3. The accuracy of the method was determined by recovery testing at three concentration levels (12.5, 25, and 50 µg/mL). The percentage recoveries of ISDN ranged from 100.05 ± 0.76% to 101.59 ± 0.54%, while those of HDZ ranged from 99.25 ± 0.32% to 101.56 ± 0.67%. These results demonstrated that the method provided accurate quantification of both ISDN and HDZ within the tested concentration range. The precision of the analytical method was evaluated by six replicate measurements of ISDN and HDZ standard solutions at 25 µg/mL. The %RSD values of peak areas were 0.96% for ISDN and 0.08% for HDZ, indicating good repeatability and precision of the HPLC method.

### 3.7. Drug Loading Reproducibility, Content Uniformity, and Drug Loss During FDCD Preparation

The reproducibility of the gel-loading process was initially evaluated in single-drug-loaded FDCDs based on the weight variation in the loaded gel formulations. The gel-loading process showed low variability, with %RSD values ranging from 1.12% to 2.21% among all ISDN and HDZ gel formulations. For the dual-drug-loaded FDCDs, drug content uniformity was evaluated by determining the actual ISDN and HDZ contents in each individual FDCD and expressing the measured drug amount as a percentage of the theoretical drug loading. The mean drug contents were 98.06 ± 0.43% for ISDN and 98.33 ± 0.18% for HDZ, with %RSD values of 0.44% and 0.19%, respectively. The effect of the drying process at 60 °C for 12 h on drug retention was evaluated by comparing the actual drug amount before and after drying. For ISDN, the drug amount decreased from 39.60 ± 0.08 mg before drying to 39.22 ± 0.17 mg after drying, corresponding to a drug loss of 0.96 ± 0.37%. For HDZ, the drug amount decreased from 37.11 ± 0.01 mg before drying to 36.87 ± 0.07 mg after drying, corresponding to a drug loss of 0.62 ± 0.22%. These results indicated that the drying process did not significantly affect the drug content of ISDN and HDZ during FDCD preparation (*p* > 0.05).

### 3.8. In Vitro Floating Ability and Dual-Drug Release Profile of ISDN3- and HDZ1-Loaded FDCDs

**Figure 6:**
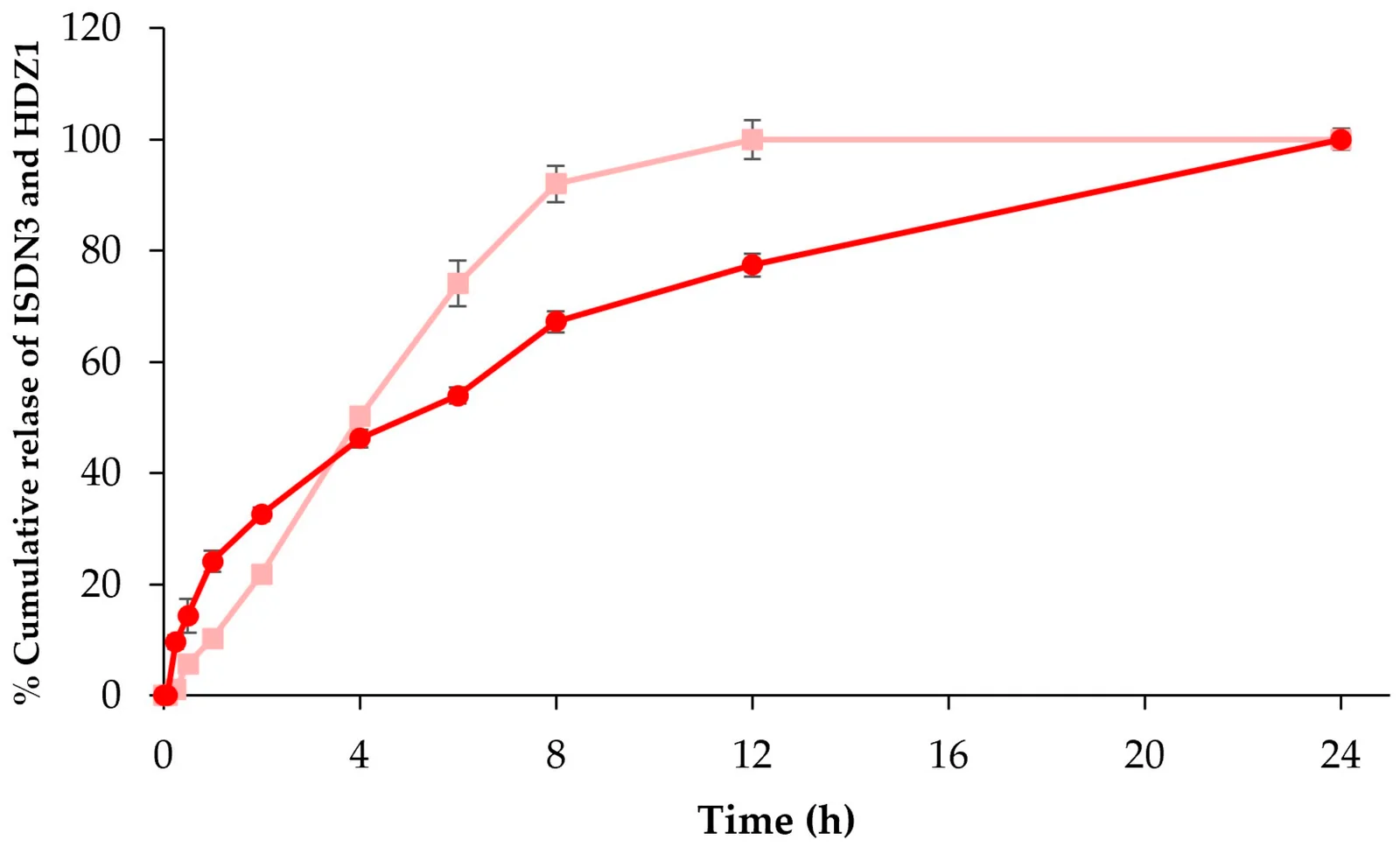
In vitro drug release of the ISDN (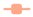) and HDZ (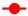) from the ISDN3- and HDZ1-loaded FDCDs. All measurements were performed with three independent devices (*n* = 3).

The dual-drug-loaded FDCDs rapidly achieved a floating state upon immersion in SGF, with a floating lag time of 0 min, and remained floating throughout the dissolution period. The in vitro release profiles of ISDN and HDZ from the ISDN3- and HDZ1-loaded FDCDs are shown in Figure 6. The dual-drug-loaded FDCDs demonstrated sustained release behavior for both drugs under SGF (pH 1.2). The ISDN release profile from the dual-drug-loaded FDCDs showed a gradual release pattern, reaching approximately 50% cumulative release within 4 h and complete release within 12 h. HDZ exhibited a relatively faster release profile, with approximately 77% cumulative release achieved within 12 h, followed by complete release at 24 h. Two-way repeated-measures ANOVA with Greenhouse–Geisser correction showed no significant main effect of loading configuration on ISDN release (*p* = 0.1158), but a significant loading configuration × time interaction was observed (*p* = 0.0447). For HDZ, both the loading configuration effect (*p* = 0.0065) and its interaction with time (*p* = 0.0010) were significant. Holm-adjusted follow-up comparisons detected no significant differences at individual sampling times for ISDN, whereas HDZ release from the dual-drug-loaded FDCDs was lower at 6 and 8 h (adjusted *p* = 0.0357 and 0.0296, respectively). Detailed statistical results are provided in Tables S5 and S6. These findings demonstrated that the FDCD system enabled simultaneous sustained release of ISDN and HDZ from separate compartments, although time-dependent differences in the release profiles were observed, particularly for HDZ.

### 3.9. Post-Dissolution Evaluation of FDCDs

The physical changes in FDCDs after the dissolution study were evaluated by visual observation, dimensional measurement, and weight measurement. No visible swelling, deformation, cracking, or erosion was observed after the dissolution period, and the FDCDs maintained their apparent structure as shown in Figure S7. No significant changes in the diameter and height of the FDCDs were observed after dissolution. In addition, the weight of the empty FDCDs before gel loading was 935.10 ± 0.40 mg, and no measurable change in device weight was observed after dissolution, removal of the residual drug gel, and oven-drying at 50 °C for 24 h. These results indicate that the FDCD shells maintained their physical integrity under the in vitro dissolution conditions.

### 3.10. Drug Release Kinetics

The release kinetics of ISDN and HDZ from single- and dual-drug-loaded FDCDs were evaluated using zero-order, first-order, and Higuchi kinetic models. The fitting intervals applied for kinetic analysis of each FDCD formulation are summarized in Table S4. These intervals were selected individually based on the principal release phase, extending from the first measurable release point to the last sampling point before a clear leveling-off of the cumulative release profile; when no plateau was observed, data up to the final available sampling point were included. The goodness of fit of each kinetic model was evaluated based on both R^2^ and RMSE values (Table 4). The most appropriate model was selected primarily based on the R^2^, while RMSE was used as a complementary measure of fitting error, particularly when R^2^ values were similar among candidate models. The selected models were interpreted solely as empirical mathematical descriptions of the release profiles and were not regarded as definitive evidence of the underlying drug release mechanisms.

For ISDN-loaded FDCDs, different release kinetic behaviors were observed depending on the gel formulation. The ISDN1 formulation was best described by the first-order model, which showed a high R^2^ value. Although the Higuchi model exhibited a slightly lower RMSE (4.01% compared with 4.92% for the first-order model), the first-order model was retained based on the predefined selection approach in which R^2^ was the primary criterion and RMSE was used as a complementary measure of fitting error. In contrast, ISDN2 and ISDN3 formulations exhibited the best fit with the zero-order model, which provided the highest R^2^ values together with relatively low RMSE values among the evaluated models.

For HDZ-loaded FDCDs, all formulations (HDZ1–HDZ3) were best described by the Higuchi model. Although the R^2^ and RMSE-based rankings were discordant for HDZ1 and HDZ2, the Higuchi model was retained according to the predefined model-selection approach. For HDZ3, both goodness-of-fit measures supported the same model selection. For the dual-drug-loaded FDCDs, the kinetic fitting results were consistent with those observed in the corresponding single-drug-loaded formulations. The ISDN3-loaded compartment was best described by the zero-order model, while the HDZ1-loaded compartment was best described by the Higuchi model. However, this similarity in mathematical fitting should not be interpreted as confirmation that the underlying drug release mechanisms remained unchanged.

## 4. Discussion

The FDCDs had a smooth surface due to the layer-by-layer extrusion in the FDM 3D printing technique [41]. In addition, the sizes (diameter, height, and compartment diameter) of the FDCDs were similar to those of the designed FDCD model. The FDM 3D printing process demonstrated high precision and minimal shape error, as indicated by the low standard deviation values of weight and dimensions. These findings are consistent with previous studies, which reported that FDM 3D printing exhibits the highest accuracy with a minimal shape error of 0.05% [42,43]. The observed white color of the FDCD was due to the PLA filament’s color. As reported in previous studies, these results indicated that the 3D printing process produces objects in the same color as the feed material [26,44]. The FDCDs were fabricated using commercial eSUN PLA+ filament. According to the manufacturer’s safety data sheet, the material is primarily composed of PLA resin with a small proportion of unspecified additives. Although eSUN PLA+ is produced from plant-based PLA materials and is described as a food-grade material by the manufacturer, it is not specifically developed as a pharmaceutical-grade material. Detailed information regarding the identity and function of the additives and pigments was not publicly disclosed by the manufacturer. Therefore, further evaluation, including extractables/leachables and biocompatibility assessments, would be required before clinical application. Regarding the potential oral administration of the FDCDs, the device dimensions were within the size range described in FDA guidance for solid oral dosage forms. Nevertheless, device dimensions alone may not fully predict swallowability and patient acceptability. Since the effect of FDCD size and geometry on these factors was not evaluated in this study, further investigation is required in future studies.

The characterization of the base gel formulations provided important insights into the influence of polymer composition on gel properties prior to incorporation into the FDCDs. The results of this study indicate that the type and concentration of polymer played an important role in determining the physical and rheological characteristics of the gel matrices. The increased PVP concentration in ISDN-base formulations and HPMC concentration in HDZ-base formulations resulted in higher apparent viscosity, which may be attributed to enhanced polymer–water interactions and increased resistance to flow within the gel matrix. The higher viscosity observed in formulations containing greater polymer concentrations suggested the formation of a more structured gel matrix. All base gel formulations exhibited shear-thinning behavior, consistent with the pseudoplastic behavior reported for PVP-containing mixed-polymer gels and concentrated HPMC hydrogels [45,46]. The decrease in viscosity under applied shear may facilitate processing and loading of the gel formulations into the FDCDs. The differences in rheological properties among formulations may influence subsequent drug release behavior by affecting medium penetration and drug diffusion through the polymer matrix. Pan et al. reported slower benzophenone-4 release from the more concentrated and viscous HPMC gel, supporting a possible relationship between gel rheology and release rate [17,46]. However, these rheological measurements were performed on drug-free base gels, and drug incorporation may alter gel viscosity and structure. The observed viscosity differences therefore provide preliminary information on the effects of polymer composition but may not fully represent the final drug-loaded formulations. Consequently, any relationship between base-gel viscosity and the observed drug release profiles should be interpreted cautiously, as these rheological measurements alone cannot establish the release mechanisms of the drug-loaded FDCDs.

The developed analytical methods demonstrated good linearity, acceptable accuracy, and precision for ISDN and HDZ quantification. The analytical methods were subsequently applied to evaluate drug content and release profiles from single-drug-loaded and dual-drug-loaded FDCDs formulations. For ISDN-loaded FDCDs, the developed HPLC method demonstrated reliable analytical performance for ISDN quantification. The ISDN peak was clearly detected at an RT of approximately 5.03 min, which was comparable with previously reported HPLC methods for ISDN determination [38]. The HDZ-loaded FDCDs were evaluated using the developed UV–visible spectrophotometric method for HDZ quantification. The method demonstrated acceptable analytical performance and was successfully applied to determine HDZ concentration during the release study of HDZ-loaded FDCDs. The developed HPLC analytical method allowed simultaneous quantification of ISDN and HDZ in the dual-drug-loaded FDCDs. The analytical performance was comparable with previous reports describing HPLC quantitative methods, which showed reliable analytical performance for the determination of pharmaceutical compounds in complex matrices [39,40]. Therefore, the developed method was considered suitable for the simultaneous quantification of ISDN and HDZ in the dual-drug-loaded FDCDs.

The high drug content uniformity observed among individual dual-drug-loaded FDCDs, as indicated by the low %RSD values for ISDN and HDZ, demonstrated the reproducibility and consistency of the gel-loading process. The uniform drug distribution within individual FDCDs may be attributed to the controlled loading of predefined amounts of drug-containing gel formulations into separate compartments of the device. In addition, the dual-compartment design enabled independent incorporation of ISDN and HDZ gel formulations, which may reduce variability associated with simultaneous loading of different drug formulations. These results indicate that the developed loading approach provided consistent drug incorporation and was suitable for the preparation of dual-drug-loaded FDCDs with reliable drug content. The minimal drug loss observed after the loading and drying processes indicated that the selected preparation conditions were appropriate for maintaining ISDN and HDZ contents within the dual-drug-loaded FDCDs. The low drug loss may be attributed to the incorporation of both drugs within the polymer-based gel matrices, which may reduce drug loss during the drying process. In addition, the drying temperature applied in this study (60 °C) was considerably lower than the reported thermal degradation temperatures of both ISDN and HDZ, which occur at temperatures above 150 °C [47,48]. Therefore, the selected drying condition was unlikely to induce significant thermal degradation of either drug, supporting the high drug retention observed after the FDCDs preparation process. These findings demonstrated that the gel loading and drying procedures were suitable for preparing dual-drug-loaded FDCDs while maintaining consistent ISDN and HDZ content. The reproducible drug loading and minimal drug loss support the applicability of the developed preparation process for achieving a reliable dual-drug delivery system.

An anti-flip-up evaluation was conducted under simulated gastric conditions to assess the positioning of the device, ensuring that the dosage form would maintain its buoyancy without overturning during the release process. The anti-flip-up of the ISDN and HDZ-loaded FDCDs prevented overturning because the FDCDs had an air chamber in the upper part of the device, resulting in immediate buoyancy in the SGF. In the previous article, Huanbutta et al. developed an anti-flip-up floating tablet fabricated by 3D printing with PLA filament. The designed model contained an air cavity in the upper part of the tablet, with an air compartment height of 2.0 mm. The results showed that the assembled tablet stably floated without flipping up during the evaluation period [49]. In addition, all formulations of ISDN- and HDZ-loaded FDCDs, which contained a 3.5 mm air cavity, exhibited immediate floating ability in SGF, with a floating lag time of 0 min, and maintained floating ability for over 24 h, because the design incorporates an air cavity of the FDCDs by non-effervescent systems using a low-density matrix mechanism, which has a density of the tablet less than gastric fluids, allowing it to exhibit in vitro floating ability in the SGF [50]. Furthermore, the PLA-based FDCDs demonstrated in vitro floating ability for more than 24 h in SGF. This behavior may be attributed to the hydrophobic characteristics of PLA [34], which help maintain the integrity of the device structure and preserve the internal air compartment under acidic conditions. Previous studies also reported a floating lag time of 0 min, which was attributed to the low-density property of the whole formulation [25,51,52]. However, in vitro floating ability does not directly confirm gastric retention in humans, as gastrointestinal residence time is influenced by physiological factors, including gastric motility, fed/fasted conditions, and gastric emptying behavior. Previous studies have provided preliminary evidence regarding the gastrointestinal transit and tolerability of PLA-based floating devices. Shin et al. reported that a 3D-printed PLA floating device containing an acyclovir sustained-release tablet remained in the stomach of Beagle dogs for more than 12 h, was observed in the small intestine at 24 h, and was no longer detected by abdominal radiography at 48 h without signs of vomiting or pain [53]. Therefore, the gastrointestinal transit, safety, and potential extractables of the PLA-based FDCD developed in this study require further in vivo evaluation.

The dissolution profiles of ISDN and HDZ commercial tablets showed immediate release within 30 min. The results were consistent with previous studies, in which ISDN commercial tablets were fully released within 45 min [36,37], and HDZ commercial tablets achieved 100% dissolution within 20 min [33]. On the other hand, the release profiles of ISDN and HDZ with different formulations containing FDCDs exhibited immediate floating with sustained release profiles at 0-24 h dissolution period under simulated gastric conditions. ISDN was completely released within 12 h, whereas HDZ demonstrated complete release within 24 h. The dissolution studies were conducted under sink conditions. Complete release of 40 mg ISDN and 37.5 mg HDZ per device into 900 mL of SGF would produce maximum theoretical concentrations of 44.44 and 41.67 µg/mL, respectively. The reported aqueous solubility values of ISDN and HDZ are 1.089 and 40 mg/mL, respectively [36]. Thus, the maximum theoretical concentrations corresponded to only 4.08% and 0.10% of the reported solubility limits of ISDN and HDZ, respectively, confirming that sink conditions were maintained. The release behavior may be attributed to the structure of the FDCDs fabricated from PLA, which could protect the drug-containing gel from direct contact with SGF, resulting in a decreased drug release rate. In addition, the constant diameter of the compartment is important for stable, sustained drug release. Moreover, the large number of compartments was significant for the fast drug release rate. The findings were consistent with earlier research, which reported that the number of holes affects the release profiles, with fewer holes resulting in slower release than a greater number of holes [26,54]. Therefore, the FDCD was designed with two compartments, resulting in a slow-release profile. Furthermore, the polymer content significantly influenced the dissolution behavior of the ISDN and HDZ formulations. For the ISDN formulations, ISDN3 containing the highest PVP ratio showed a slower release rate compared with ISDN1 and ISDN2. This behavior may be attributed to the formation of a more viscous gel matrix, which could restrict water penetration and retard drug diffusion from the formulation. In contrast, the lower ratios of PVP in ISDN1 and ISDN2 resulted in the fastest dissolution. PVP is a highly water-soluble polymer with excellent biocompatibility [55], and its rapid dissolution was mainly attributed to the pyrrolidone functional group (lactam ring), which contains a carbonyl group (C=O) and a nitrogen atom capable of forming hydrogen bonds with the medium. This interaction allows PVP to dissolve quickly in the stomach fluid [56,57]. Furthermore, the formulation of HDZ1 was successful, with 100% release at 24 h, while HDZ2 and HDZ3 did not achieve release within 24 h. HDZ1 had a lower HPMC concentration, leading to complete release at 24 h. HPMC is a hydrophilic polymer that forms a viscous gel layer upon hydration. The swelling and gelation of HPMC can lead to more sustained and controlled drug release [58,59]. Therefore, the combination of HPMC and PVP concentrations in the gel formulations was an important factor in controlling the release behavior of HDZ and achieving the desired release profile. ISDN and HDZ have been reported as biopharmaceutics classification system (BCS) class III drugs, characterized by high aqueous solubility and low permeability [36,37]. Therefore, drug dissolution was considered unlikely to be the primary factor controlling the release behavior under the investigated conditions. Instead, the release behavior was mainly governed by the polymer matrix properties, including hydration, gel formation, and diffusion resistance. The selection of HPMC and PVP polymers contributed to tailoring the release profiles of the individual drug formulations, resulting in approximately 12 h release for ISDN and 24 h release for HDZ. The developed FDCDs provided a platform for achieving controlled release of ISDN and HDZ from a single device. Trivedi et al. developed a bilayer drug tablet with immediate release of HDZ and sustained release of ISDN, prepared using the direct compression method. The bilayer tablet exhibited HDZ, achieving 100% release within 30 min in SGF, while ISDN showed 91.67% release at 6 h in simulated intestinal fluid (SIF) [60]. In contrast, the FDCD developed in this study enabled sustained release of both drugs under simulated gastric conditions, with ISDN reaching complete release within 12 h and HDZ within 24 h. In addition, the ISDN3-loaded FDCD exhibited a sustained-release duration comparable to the reported release duration of commercially available modified-release ISDN tablets. Cardiket Retard 40 mg has been reported to achieve approximately 90% drug release over 12 h [61]. However, Cardiket Retard was not used as a direct comparator in this study, and differences in dissolution conditions prevent direct comparison of the complete release profiles. For HDZ, Saquib et al. developed a floating-bioadhesive tablet for the gastroretentive release of HDZ using the direct compression method. The results presented tablets that could float for more than 12 h in SGF with a lag time of less than 35 s and showed a well-bioadhered time of more than 4 h on intestinal mucosa in SGF; the release demonstrated sustained release for more than 12 h [62]. Recent advances in FDM 3D printing have enabled the development of floating multidrug delivery systems. Zgouro et al. reported a floating 3D-printed polypill using an HPMC-based filament containing diltiazem, propranolol, and hydrochlorothiazide, demonstrating sustained release of multiple drugs from a polymeric matrix system [63]. In contrast, the present FDCD platform utilized a preprinted PLA device with separately loaded ISDN and HDZ gel formulations in independent compartments. This design separates the fabrication of the device structure from drug incorporation, thereby avoiding direct exposure of the drug formulations to the high-temperature extrusion process during FDM printing. Moreover, the compartmentalized drug-loading strategy allows different polymer systems to be applied for each drug according to their formulation requirements. In the present study, PVP-based ISDN gel and PVP/HPMC-based HDZ gel were independently optimized to achieve different sustained-release profiles within a single device. Therefore, the potential advantage of the present FDCD platform lies in its flexibility for drug-specific formulation optimization and independent modulation of drug release behavior, rather than the introduction of 3D-printed multidrug delivery technology itself.

The dual-drug-loaded FDCDs maintained sustained release of both drugs, with ISDN3 reaching complete release within 12 h and HDZ1 achieving complete release within 24 h. However, the statistical comparison showed that the release profiles were not fully equivalent to those of the corresponding single-drug-loaded FDCDs. In particular, HDZ showed a significant loading configuration effect and a significant loading configuration × time interaction, with lower release from the dual-drug-loaded FDCDs at 6 and 8 h. ISDN also showed a significant loading configuration × time interaction, although no significant overall effect of loading configuration or time-specific differences were detected. Thus, similar release completion times should not be interpreted as evidence of equivalent release profiles. The difference in release duration between ISDN and HDZ was mainly attributed to the different physicochemical properties and hydration behaviors of the polymer matrices. Although ISDN3 contained a higher concentration of PVP, direct comparison of release behavior based solely on viscosity is inappropriate because PVP and HPMC exhibit different hydration and dissolution characteristics. PVP is a highly water-soluble polymer that readily dissolves upon contact with aqueous media, which may facilitate drug diffusion from the polymer matrix [64]. In contrast, HPMC undergoes hydration and swelling to form a gel layer that acts as a diffusion barrier, thereby controlling drug release through restricted water penetration and drug diffusion [58,65,66]. Therefore, the longer release duration of HDZ1 compared with ISDN3 was likely associated with the gel-forming and swelling properties of HPMC rather than polymer viscosity alone. These findings indicated that loading ISDN3 and HDZ1 gels into separate compartments of the same FDCD enabled sustained release of both drugs. Overall, loading ISDN3 and HDZ1 gels into separate compartments of the same FDCD enabled sustained release of both drugs, although time-dependent differences in release behavior were observed after incorporation into the dual-drug system.

The drug loading doses of the dual-drug-loaded FDCDs were selected based on the marketed fixed-dose ISDN/HDZ combination product (BiDil^®^), which contains 20 mg of ISDN and 37.5 mg of HDZ per immediate-release tablet. According to the FDA-approved prescribing information, the recommended starting dose of BiDil^®^ is one tablet three times daily, with titration up to two tablets three times daily based on patient tolerance. In addition, clinical studies have reported an initial dosing regimen of one tablet twice daily followed by dose titration according to patient tolerance and clinical response [32]. Considering these dosing regimens, the FDCD was designed to incorporate 40 mg of ISDN, corresponding to the total ISDN amount administered during the initial twice-daily clinical regimen, while maintaining HDZ loading at 37.5 mg, equivalent to the marketed product strength. This design aimed to provide sustained release of both drugs using drug amounts selected based on the marketed product strength, reported clinical dosing range, and formulation feasibility. However, the selected drug loading does not represent a direct dose equivalence to the marketed immediate-release BiDil^®^ formulation or a complete twice-daily BiDil^®^ regimen, because the FDCD was developed as a sustained-release delivery platform. The current FDCD should therefore be considered a proof-of-concept sustained-release platform rather than a direct replacement for the approved BiDil^®^ regimen. In addition, prolonged continuous exposure to nitrates may potentially contribute to nitrate tolerance. Therefore, the design of sustained ISDN delivery should consider the balance between prolonged drug exposure and the need for an appropriate nitrate-free or nitrate-low interval, which requires further pharmacokinetic and pharmacodynamic investigation. Further optimization of the FDCD design, such as increasing compartment volume or incorporating additional drug-loading compartments, may enable higher drug loading. However, these modifications may increase the overall device size, which should be further evaluated with respect to swallowability and patient acceptability. Despite the limitation of the current FDCD design in terms of drug-loading capacity, the selected drug amounts were within the reported clinical dosing range and were intended to achieve the desired sustained-release profiles. Compared with conventional immediate-release tablets, the FDCD provides a modified release system that may influence drug exposure over time. Previous studies have reported that floating drug delivery systems fabricated using FDM 3D printing can influence pharmacokinetic behavior through prolonged gastric residence time and controlled drug release. Chai et al. reported an in vivo pharmacokinetic study of an FDM 3D-printed floating tablet containing domperidone, in which the floating and conventional immediate-release tablets were administered at an equivalent drug dose. The floating tablet demonstrated significantly higher systemic exposure than the conventional immediate-release tablet, suggesting that prolonged gastric retention and controlled drug release may influence pharmacokinetic behavior in specific systems [67]. However, the pharmacokinetic behavior of the developed FDCD remains to be confirmed through further in vivo studies.

Several mathematical models have been applied to describe drug release profiles from FDCD systems. In this study, the best-fitting model was selected primarily based on the R^2^ value, while RMSE was used as a complementary measure of fitting error, particularly when R^2^ values were similar among candidate models. Therefore, the selected model represents the best empirical mathematical fit rather than definitive confirmation of the release mechanism.

For ISDN-loaded FDCDs, based primarily on the R^2^ values, ISDN1 showed the best fit with the first-order model, whereas ISDN2 and ISDN3 showed the best fit with the zero-order model. The different kinetic fitting behaviors may be related to variations in PVP concentration and gel matrix properties. Higher PVP concentrations may increase polymer viscosity and resistance to drug transport, contributing to prolonged drug release [68,69,70].

For all HDZ-loaded FDCD formulations, the Higuchi model provided the best empirical fit based primarily on the R^2^ values. This behavior may be associated with the hydration and swelling properties of HPMC, which forms a hydrated gel matrix that influences drug transport through the polymer network [71,72]. Consistent with this finding, Samanthula et al. reported that the Higuchi model provided the best fit for HDZ hydrochloride release from HPMC-based buccal tablets [73].

In the dual-drug-loaded FDCDs, the ISDN3 and HDZ1 compartments maintained kinetic fitting behaviors similar to those of their corresponding single-drug-loaded FDCDs, with ISDN showing the best fit to the zero-order model and HDZ showing the best fit with the Higuchi model, based primarily on the R^2^ values. These results suggest that incorporation of both gel formulations into a single dual-compartment FDCD preserved the overall kinetic fitting patterns; however, the release profiles were not fully equivalent, as indicated by the loading configuration effect observed for HDZ and the time-dependent interactions identified for both drugs.

Beyond the individual release profiles of ISDN and HDZ, the combination of both drugs within a single FDCD provides a platform for simultaneous delivery of two complementary cardiovascular agents. ISDN and HDZ have different pharmacological actions, with ISDN primarily acting as a venous vasodilator and HDZ functioning as an arterial vasodilator. Therefore, the developed dual-drug FDCDs may provide an approach for further investigation of combined drug delivery for heart failure treatment.

## 5. Conclusions

In this study, we successfully manufactured FDCDs by FDM 3D printing using PLA filament, containing a dual cardiovascular drug gel to modify drug release rates *via* various formulations. The FDCD was designed in a cylindrical shape with floating properties, utilizing a non-effervescent system with a low-density air chamber and two compartments to contain the drug gels. The developed FDCDs exhibited immediate floating, resistance to overturning, and in vitro floating ability under simulated gastric conditions. The ISDN3- and HDZ1-loaded FDCDs demonstrated sustained release profiles, achieving complete release of ISDN within 12 h and HDZ within 24 h, with the release profiles showing the highest correlation with the zero-order model for ISDN3 and the Higuchi model for HDZ1. These findings demonstrate the potential of the dual-drug-loaded FDCDs as a formulation for simultaneous delivery of ISDN and HDZ with sustained release characteristics. Further in vivo studies are required to evaluate gastrointestinal transit, safety, pharmacokinetic performance, and therapeutic outcomes of the developed systems.

## Figures and Tables

**Table 1:** Composition of base gel formulations used for rheological characterization.

Formulations	Ratios		
	PVP (g)	HPMC (g)	DI Water (g)
ISDN1-base	3.0	-	5.0
ISDN2-base	4.0	-	4.0
ISDN3-base	5.0	-	3.0
HDZ1-base	2.5	0.3	5.2
HDZ2-base	2.5	1.0	4.5
HDZ3-base	2.5	1.75	3.75

**Table 2:** Formulations of ISDN and HDZ gels.

Formulations	Ratios			
	Drug (g)	PVP (g)	HPMC (g)	DI Water (g)
ISDN1	2.0	3.0	-	5.0
ISDN2	2.0	4.0	-	4.0
ISDN3	2.0	5.0	-	3.0
HDZ1	2.0	2.5	0.3	5.2
HDZ2	2.0	2.5	1.0	4.5
HDZ3	2.0	2.5	1.75	3.75

**Table 3:** Analytical performance parameters of the HPLC method for simultaneous quantification of ISDN and HDZ.

Parameters		ISDN	HDZ
Linearity	Concentration range (µg/mL)	1.56–100	1.56–100
	R^2^	0.9993	0.9999
	Slope of calibration curve	25.684	226.78
	Intercept of calibration curve	13.772	101.52
Accuracy (%Recovery)	12.5 µg/mL	100.05 ± 0.76	101.56 ± 0.67
	25 µg/mL	101.37 ± 0.87	99.25 ± 0.32
	50 µg/mL	101.59 ± 0.54	99.38 ± 0.23
Precision (%RSD)	25 µg/mL	0.96	0.08

**Table 4:** Drug release kinetics of ISDN and HDZ from single- and dual-drug-loaded FDCDs.

Formulations	Model	R^2^	RMSE (%)	Fitted Equation	Fitted Parameter
ISDN1-loaded FDCDs	Zero-order	0.9124	9.28	y = 14.454x + 20.86	*k*_0_ = 14.45
	First-order *	0.9873	4.92	y = −0.6075x + 4.7132	*k*_1_ = 0.61
	Higuchi	0.9837	4.01	y = 44.813x − 6.297	*k_H_* = 44.81
ISDN2-loaded FDCDs	Zero-order *	0.9859	4.87	y = 15.061x + 5.7615	*k*_0_ = 15.06
	First-order	0.9679	15.33	y = −0.4077x + 4.763	*k*_1_ = 0.41
	Higuchi	0.9546	29.82	y = 38.300x − 25.111	*k_H_* = 38.30
ISDN3-loaded FDCDs	Zero-order *	0.9901	3.90	y = 12.107x + 3.0709	*k*_0_ = 12.11
	First-order	0.9409	24.69	y = −0.3496x + 4.8367	*k*_1_ = 0.35
	Higuchi	0.9707	23.56	y = 36.409x − 17.465	*k_H_* = 36.41
HDZ1-loaded FDCDs	Zero-order	0.9202	5.55	y = 2.4138x + 10.064	*k*_0_ = 2.41
	First-order	0.9785	3.44	y = −0.0392x + 4.5236	*k*_1_ = 0.04
	Higuchi *	0.9907	4.87	y = 14.119x − 5.0801	*k_H_* = 14.12
HDZ2-loaded FDCDs	Zero-order	0.9685	4.08	y = 2.9007x + 8.854	*k*_0_ = 2.90
	First-order	0.9961	7.68	y = −0.0548x + 4.5725	*k*_1_ = 0.05
	Higuchi *	0.9974	6.17	y = 16.552x − 8.379	*k_H_* = 16.55
HDZ3-loaded FDCDs	Zero-order	0.8503	10.97	y = 3.712x + 24.32	*k*_0_ = 3.71
	First-order	0.9714	7.77	y = −0.1804x + 4.6272	*k*_1_ = 0.18
	Higuchi *	0.9835	7.64	y = 21.29x + 3.5899	*k_H_* = 21.29
Dual-drug-loaded FDCDs (ISDN3)	Zero-order *	0.9960	2.12	y = 12.019x − 0.8963	*k*_0_ = 12.02
	First-order	0.9391	9.01	y = −0.2935x + 4.781	*k*_1_ = 0.29
	Higuchi	0.9748	5.30	y = 39.87x − 25.63	*k_H_* = 39.87
Dual-drug-loaded FDCDs (HDZ1)	Zero-order	0.8749	7.13	y = 3.7039x + 23.517	*k*_0_ = 3.70
	First-order	0.9573	5.86	y = −0.1794x + 4.697	*k*_1_ = 0.18
	Higuchi *	0.9884	3.47	y = 21.014x + 2.3098	*k_H_* = 21.01

*Q_t_*, cumulative percentage of drug released at time *t* (%); *t*, release time (h); *k*_0_, zero-order release constant (% h^−1^); *k*_1,_ first-order release constant (h^−1^); *k_H,_* Higuchi release constant (% h^−1/2^). For the zero-order model, *Q_t_* was plotted against *t*; for the first-order model, ln(100 − *Q_t_*) was plotted against *t*; and for the Higuchi model, *Q_t_* was plotted against *t*^1/2^. RMSE is expressed as a percentage of cumulative drug release. * Indicates the selected empirical best-fitting kinetic model. Model selection was based primarily on the R^2^, while RMSE was used as a complementary measure of fitting error, particularly when R^2^ values were similar among candidate models. The selected models were interpreted as empirical mathematical descriptions of the release profiles and not as confirmation of the underlying drug release mechanisms.

## Data Availability

The data supporting the findings of this study are available in the article and its supplementary data.

## Supplementary Materials

The following supporting information can be downloaded at: https://www.mdpi.com/article/10.3390/pharmaceutics18101278/s1.

## Abbreviations

The following abbreviations are used in this manuscript:

GRFDDS	Gastroretentive floating drug delivery system
3D	Three-dimensional
FDA	Food and Drug Administration
FDM	Fused deposition modeling
PVA	Polyvinyl alcohol
PLA	Polylactic acid
ISDN	Isosorbide dinitrate
HDZ	Hydralazine
ACEIs	Angiotensin-converting enzyme inhibitors
ARBs	Angiotensin II receptor blockers
FDCD	Floating dual-compartment device
GPO	Government pharmaceutical organization
NaCl	Sodium chloride
HPMC	Hydroxypropyl methylcellulose
PVP K-90	Polyvinyl pyrrolidone
SD	Standard deviation
DI	Deionized
SGF	Simulated gastric fluid
HPLC	High-performance liquid chromatography
R^2^	Coefficient of determination
RSD	Relative standard deviation
RMSE	Root mean square error
RT	Retention time
BCS	Biopharmaceutics classification system
