# Supplementary material for: 3D-Printed Gastroretentive Floating Modified-Release Device for Dual Cardiovascular Drug Delivery: Isosorbide Dinitrate and Hydralazine
Source: Pharmaceutics. 2026 Oct 9;18(10):1278. doi: 10.3390/pharmaceutics18101278 (PMC13708162; doi:10.3390/pharmaceutics18101278)
Supplement: Supplementary file 1 [file pharmaceutics-18-01278-s001.zip › pharmaceutics-4529460-supplementary.pdf]

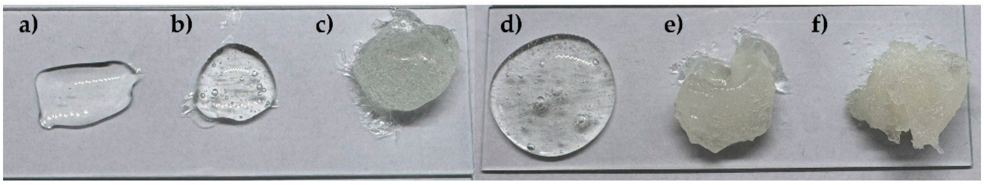

Supplementary Figure S1. Physical appearance of ISDN and HDZ base gel formulations. (a–c) ISDN1–ISDN3 formulations; (d–f) HDZ1–HDZ3 formulations.

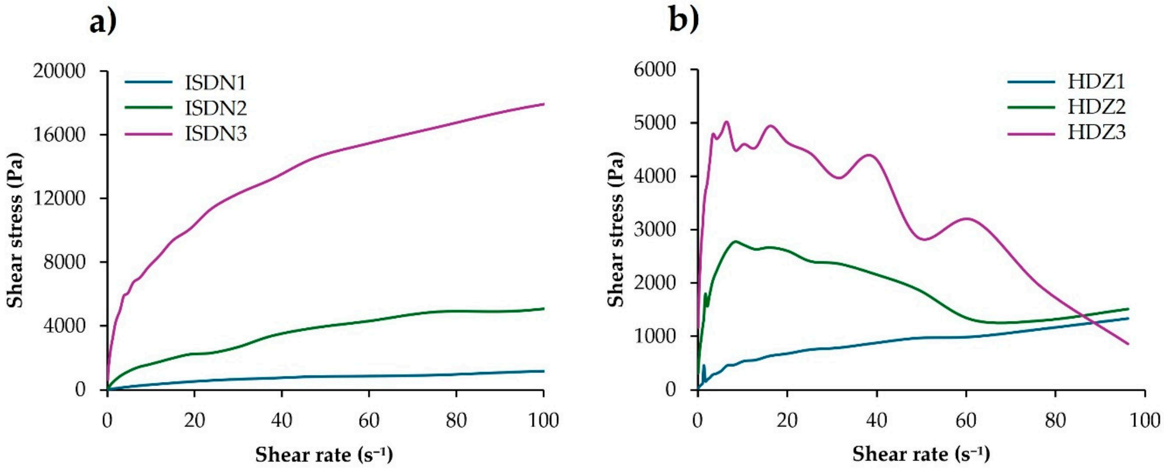

Supplementary Figure S2. Shear stress versus shear rate profiles of (a) ISDN and (b) HDZ base gel formulations.

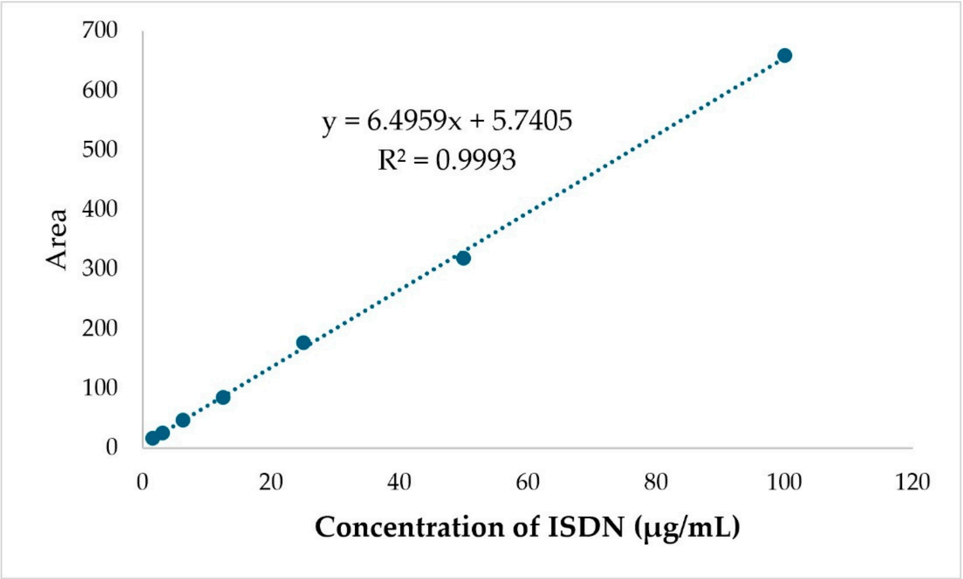

Supplementary Figure S3. Calibration curve of ISDN in SGF (0.1 N HCl) at pH 1.2 determined using HPLC at 214 nm. Each point represents the mean absorbance of triplicate measurements (n = 3).

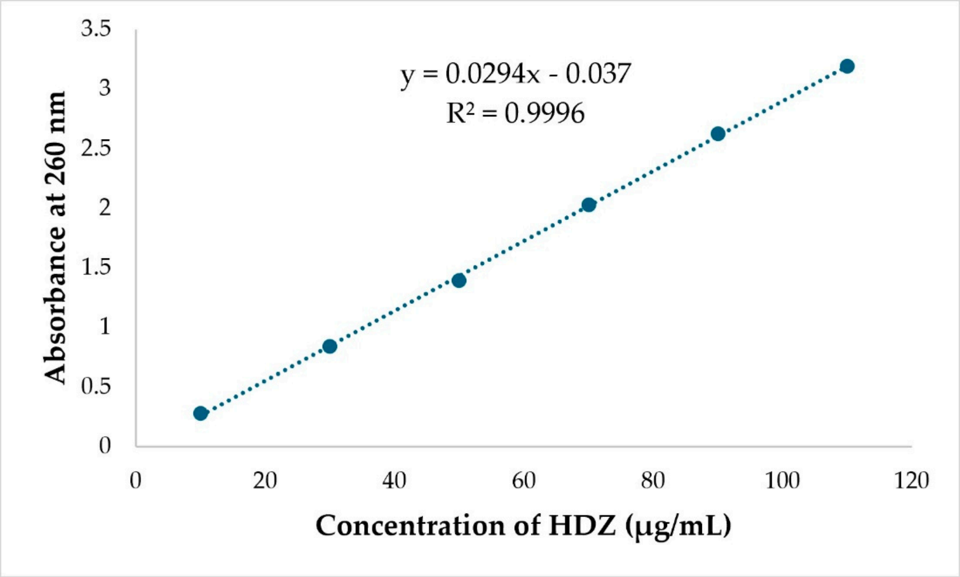

Supplementary Figure S4. Calibration curve of HDZ in SGF (0.1 N HCl) at pH 1.2 determined using UV–vis spectrophotometry at 260 nm. Each point represents the mean absorbance of triplicate measurements (n = 3).

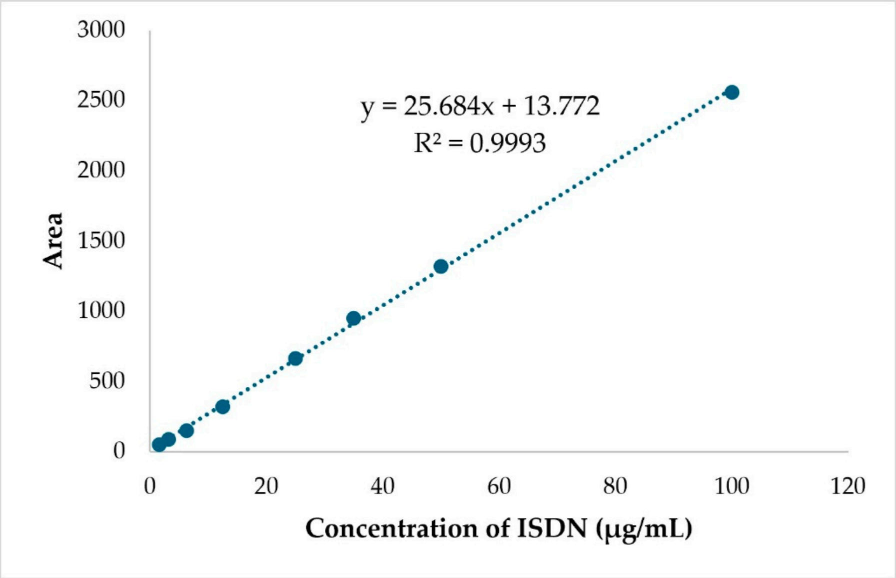

Supplementary Figure S5. Calibration curve of ISDN in SGF (0.1 N HCl) at pH 1.2 at 214 nm. Each point represents the mean area of triplicate measurements (n = 3).

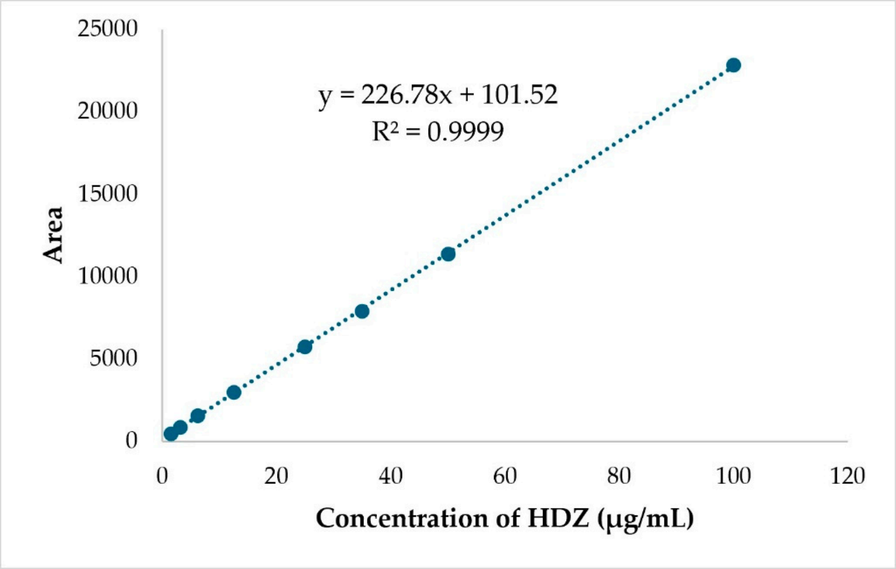

Supplementary Figure S6. Calibration curve of HDZ in SGF (0.1 N HCl) at pH 1.2 at 260 nm. Each point represents the mean area of triplicate measurements (n = 3).

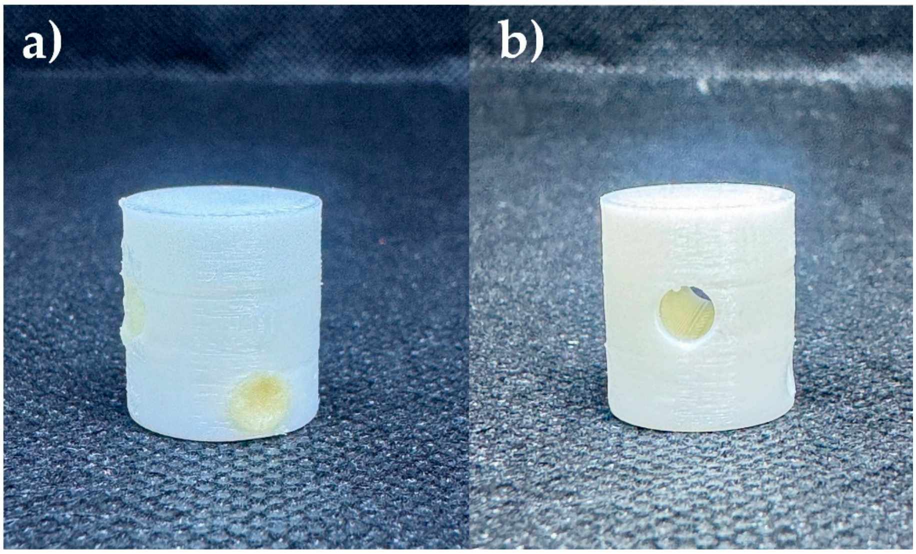

Supplementary Figure S7. Photographs of FDCDs before (a) and after (b) the dissolution study.

Supplementary Table S1. Analytical evaluation parameters of the HPLC method for ISDN quantification.

**Parameters**		**ISDN**
Linearity	Concentration range (μg/mL)	1.56–100
	R^2	0.9993
	Slope of calibration curve	6.4959
	Intercept of calibration curve	5.7405
Accuracy (%Recovery)	12.5 μg/mL	99.08 ± 1.25
	25 μg/mL	100.88 ± 0.46
	50 μg/mL	100.39 ± 0.67
Precision (%RSD)	25 μg/mL	0.59

Supplementary Table S2. Analytical evaluation parameters of the UV spectrophotometric method for HDZ quantification.

**Parameters**		**HDZ**
Linearity	Concentration range (μg/mL)	10–110
	R^2	0.9996
	Slope of calibration curve	0.0294
	Intercept of calibration curve	- 0.037
Accuracy (%Recovery)	50 μg/mL	100.66 ± 0.16
	30 μg/mL	99.24 ± 0.05
	10 μg/mL	101.25 ± 0.89
Precision (%RSD)	30 μg/mL	0.42

Supplementary Table S3. Gradient elution program of the mobile phase for the simultaneous quantification of ISDN and HDZ by HPLC.

**Time (min)**	**0.01 M Ammonium acetate buffer (pH 3) (%)**	**Acetonitrile (%)**	**Methanol (%)**
0	70	18	12
4	70	18	12
5	50	30	20
15	50	30	20
16	70	18	12
23	70	18	12

Supplementary Table S4. Fitting intervals applied for kinetic analysis of single- and dual-drug release from FDCDs.

Formulations	Fitting start time (h)	Fitting end time (h)
ISDN1-loaded FDCDs	0.25	6
ISDN2-loaded FDCDs	0.25	6
ISDN3-loaded FDCDs	0.25	8
HDZ1-loaded FDCDs	0.25	24
HDZ2-loaded FDCDs	0.25	24
HDZ3-loaded FDCDs	0.25	24
Dual-drug-loaded FDCDs (ISDN3)	0.25	8
Dual-drug-loaded FDCDs (HDZ1)	0.25	24

The fitting interval for each formulation was selected based on the principal release phase, beginning at the first measurable release point and extending to the last sampling point before a clear leveling-off of the cumulative release profile. When no plateau was observed within the experimental period, data up to the final available sampling point were included.

Supplementary Table S5. Comparison of release profiles between single- and dual-drug-loaded FDCDs.

Drug	Effect	F (df_1, df_2)	*p*-value
ISDN3	Loading configuration	4.011 (1, 4)	0.1158
ISDN3	Loading configuration × time	5.045 (1.809, 7.237)	0.0447
HDZ1	Loading configuration	27.148 (1, 4)	0.0065
HDZ1	Loading configuration × time	11.427 (2.852, 11.406)	0.001016

A two-way repeated-measures ANOVA was performed over 0.083–24 h, with loading configuration as the between-device factor and time as the within-device factor. Greenhouse–Geisser correction was applied to the interaction effect. Bold values indicate $p < 0.05$.

Supplementary Table S6. Adjusted p-values for time-specific comparisons between single- and dual-drug-loaded FDCDs.

Time (h)	ISDN: Holm-adjusted *p*	HDZ: Holm-adjusted *p*
0.083	—	—
0.25	0.6235	0.0709
0.5	0.8261	0.9219
1	0.4375	0.6153
2	0.5088	0.0643
4	0.0892	0.1005
6	0.8261	0.0357
8	0.6235	0.0296
12	—	0.0862
24	—	0.6794

Two-sided Welch's t-tests were performed with Holm adjustment separately for each drug: seven comparisons for ISDN and nine comparisons for HDZ. No post hoc comparisons were performed at time points where all observations were identical within and between groups. Bold values indicate adjusted $p < 0.05$; HDZ release was lower in the dual-drug group at 6 and 8 h.
